# Editorial: Recent advances in the assessment and management of thoracic trauma

**DOI:** 10.3389/fsurg.2023.1325928

**Published:** 2023-11-15

**Authors:** Savvas Lampridis, Marco Scarci

**Affiliations:** Department of Cardiothoracic Surgery, Hammersmith Hospital, Imperial College Healthcare NHS Trust, London, United Kingdom

**Keywords:** chest injury, chest trauma, rib fixation, rib fracture, thoracic injury, thoracic trauma

**Editorial on the Research Topic** Recent advances in the assessment and management of thoracic trauma

## Introduction

Thoracic trauma presents a significant global public health challenge, comprising approximately 25% of all injuries and contributing to up to 50% of trauma-related fatalities (1). Survivors often require extended hospitalisation and are at risk of enduring long-term disabilities. These factors highlight the urgent imperative for comprehensive strategies to address this critical issue. Despite considerable progress over recent decades, our grasp of the most effective methods for assessing and managing thoracic trauma remains hindered by crucial knowledge gaps. In light of this, the current Research Topic was developed with a primary emphasis on showcasing recent advancements in the assessment and management of thoracic injuries. The studies featured in this collection signify promising steps towards improving outcomes for patients affected by thoracic trauma. This editorial article aims to highlight key findings, acknowledge limitations, and identify avenues for future research.

## Summary of major contributions

The majority of the articles included in this compilation scrutinized outcomes following surgical stabilisation of rib fractures in distinct patient cohorts. A noteworthy endeavour was carried out by the NEXT study group, encompassing a prospective cohort study that spanned six institutions across Switzerland and the Netherlands (Hoepelman et al.). This initiative aimed to delve into the clinical outcomes and quality of life experienced by individuals after surgical rib fixation for flail chest injuries. The findings showed a significant trend, with low complication rates and a favourable one-year quality of life, as assessed with the EQ-5D-5l questionnaire. Nonetheless, it is pertinent to note that substantial variability was observed among patients in this context.

Zhang et al. embarked on an evaluation of outcomes following surgical stabilisation of rib fractures in elderly patients. Employing a single-centre, propensity score matching analysis, their investigation identified a nuanced dichotomy. Patients who underwent surgery exhibited a modestly prolonged hospital stay by an average of two days when compared with their counterparts receiving conservative treatment. Nevertheless, the surgical cohort demonstrated markedly improved rates of fracture healing and a shorter duration of analgesic regimens, delineating a balance between hospitalisation duration and therapeutic efficacy.

Becker et al. contributed a matched pairs analysis by using data from the German trauma registry. Their study focused on determining the optimal timing for rib fracture surgery. The findings highlighted a distinct advantage for surgical stabilisation within the initial 48 h post-trauma, resulting in significantly shorter durations of intensive care unit and hospital stays compared to fixation performed between three and ten days following the injury.

Shifting our attention to the study by van Veelen et al., a unique perspective emerged as the authors explored outcomes after surgical fixation of chest wall fractures incurred during cardiopulmonary resuscitation efforts. It is worth mentioning that this retrospective, single-centre study included 19 patients, representing the largest reported cohort to undergo fracture fixation due to cardiopulmonary resuscitation. The authors reported a lack of complications associated with rib fixation, with only one infection observed following sternal fixation. Furthermore, the long-term follow-up demonstrated a favourable quality of life, as measured with the EQ-5D-5l questionnaire.

## Limitations

While the studies included in this Research Topic make substantial contributions to the evidence base, it is essential to acknowledge certain limitations. In the domain of thoracic trauma, the predominant body of research is characterized by retrospective observational studies and non-randomized prospective trials. Moreover, there is a pronounced heterogeneity in surgical techniques and outcome reporting, making comparisons between studies a challenging endeavour (2). Regarding the studies in this Research Topic specifically, many of them had relatively small sample sizes, and some lacked control groups, thereby preventing comparative effectiveness analyses. Additional limitations were the single-centre design of certain studies and potential selection bias in operative cases. Furthermore, medical management and rehabilitation protocols were often not standardised or detailed. Lastly, the issue of cost-effectiveness in various treatment approaches was not thoroughly investigated.

## Future directions

While the studies featured in this Research Topic undeniably advance our understanding of the assessment and management of thoracic trauma, they also reveal a multitude of unanswered questions. Key areas requiring further research include the definition of optimal patient selection criteria for surgical stabilisation of rib fractures. Currently, it remains uncertain whether fixation should be extended to include a broader spectrum of non-flail chest fracture patterns. There is also a pressing need for comparative effectiveness studies examining different surgical techniques. The role of thoracoscopic visualization in fracture management is an interesting topic that also requires more comprehensive evaluation. Moreover, the absence of standardized protocols for perioperative analgesia and rehabilitation is conspicuous. In addition, there is a dearth of research into long-term, patient-centred outcomes, such as persistent disability and return to work. Formal cost-effectiveness analyses comparing surgical stabilisation to non-operative management are also relatively absent. To address these knowledge gaps effectively, it is necessary to conduct high-quality randomised controlled trials with standardised treatment protocols and well-defined outcomes. Multicentre studies can facilitate larger sample sizes, leading to improved generalizability. Overall, continued research is crucial in honing best practices for thoracic trauma care and ultimately enhancing outcomes for these patients. The studies presented in this Research Topic are indeed promising strides forward, but they highlight that a plethora of unanswered questions awaits exploration in future research.

## Conclusions

Thoracic trauma continues to exact a substantial toll on global health, contributing significantly to morbidity and mortality rates. The research shared in this Topic signifies prominent advancements, yet further investigations are imperative to delineate optimal practices more precisely. Persistent research efforts in this pivotal domain are warranted to mitigate the global public health repercussions of chest injuries and ultimately improve patient outcomes.
